# Scaling Interventions to Manage Chronic Disease: Innovative Methods at the Intersection of Health Policy Research and Implementation Science

**DOI:** 10.1007/s11121-022-01427-8

**Published:** 2022-09-01

**Authors:** Emma E. McGinty, Nicholas J. Seewald, Sachini Bandara, Magdalena Cerdá, Gail L. Daumit, Matthew D. Eisenberg, Beth Ann Griffin, Tak Igusa, John W. Jackson, Alene Kennedy-Hendricks, Jill Marsteller, Edward J. Miech, Jonathan Purtle, Ian Schmid, Megan S. Schuler, Christina T. Yuan, Elizabeth A. Stuart

**Affiliations:** 1 Department of Health Policy and Management, Johns Hopkins Bloomberg School of Public Health, Baltimore, MD, USA; 2 Department of Mental Health, Johns Hopkins Bloomberg School of Public Health, Baltimore, MD, USA; 3 Department of Population Health, New York University Grossman School of Medicine, New York, NY, USA; 4 Division of General Internal Medicine, Johns Hopkins School of Medicine, Baltimore, MD, USA; 5 RAND Corporation, Washington, DC, USA; 6 Department of Engineering, Johns Hopkins University, Baltimore, MD, USA; 7 Department of Epidemiology, Johns Hopkins Bloomberg School of Public Health, Baltimore, MD, USA; 8 Indiana University School of Medicine, Indianapolis, USA; 9 Department of Public Health Policy and Management, New York University School of Global Public Health, New York City, New York, USA

**Keywords:** Policy, Implementation, Scale-up

## Abstract

Policy implementation is a key component of scaling effective chronic disease prevention and management interventions. Policy can support scale-up by mandating or incentivizing intervention adoption, but enacting a policy is only the first step. Fully implementing a policy designed to facilitate implementation of health interventions often requires a range of accompanying implementation structures, like health IT systems, and implementation strategies, like training. Decision makers need to know what policies can support intervention adoption *and* how to implement those policies, but to date research on policy implementation is limited and innovative methodological approaches are needed. In December 2021, the Johns Hopkins ALACRITY Center for Health and Longevity in Mental Illness and the Johns Hopkins Center for Mental Health and Addiction Policy convened a forum of research experts to discuss approaches for studying policy implementation. In this report, we summarize the ideas that came out of the forum. First, we describe a motivating example focused on an Affordable Care Act Medicaid health home waiver policy used by some US states to support scale-up of an evidence-based integrated care model shown in clinical trials to improve cardiovascular care for people with serious mental illness. Second, we define key policy implementation components including structures, strategies, and outcomes. Third, we provide an overview of descriptive, predictive and associational, and causal approaches that can be used to study policy implementation. We conclude with discussion of priorities for methodological innovations in policy implementation research, with three key areas identified by forum experts: effect modification methods for making causal inferences about how policies’ effects on outcomes vary based on implementation structures/strategies; causal mediation approaches for studying policy implementation mechanisms; and characterizing uncertainty in systems science models. We conclude with discussion of overarching methods considerations for studying policy implementation, including measurement of policy implementation, strategies for studying the role of context in policy implementation, and the importance of considering when establishing causality is the goal of policy implementation research.

Policy implementation is a critical component of scaling effective chronic disease prevention and management interventions. Policies can support scale-up directly by mandating, incentivizing, or promoting intervention adoption or indirectly by shaping health system environments that support adoption of innovations. However, putting a policy “on the books”—through legislation, regulation, or rulemaking at the health system or organization level—is only the first step. Fully implementing a policy designed to facilitate implementation of health interventions often requires a range of activities such as staffing, training, coaching, and performance monitoring and feedback (Fixsen et al., 2009). Decision makers need to know what policies can support intervention adoption *and* how to implement those policies. However, most policy evaluation research focuses on estimating the effect of having versus not having a policy on outcomes, ignoring questions related to the effects of implementation. The growing field of implementation science has only recently begun to consider policy implementation (Emmons & Chambers, 2021; Hoagwood et al., 2020). In this article, we discuss research methods for bridging this gap, with a motivating example focused on scaling-up an evidence-based chronic disease care management model for people with serious mental illness (SMI).

In December 2021, the Johns Hopkins ALACRITY Center for Health and Longevity in Mental Illness, which studies strategies to improve physical health among people with SMI, and the Johns Hopkins Center for Mental Health and Addiction Policy, which studies behavioral health policy, co-hosted an expert forum on approaches for studying policy implementation. This forum, which brought together researchers with expertise in health policy research, implementation science, statistics, and epidemiology, sought to identify methods for advancing the study of policy implementation. This article summarizes our group’s ideas.

The remainder of the piece is organized in five sections. First, we describe a motivating example. Second, we describe the expert forum’s objective and provide key definitions. Third, we provide an overview of approaches that can be used to study policy implementation. Fourth, we describe priorities for methodological innovations in policy implementation research identified by our group. Fifth, we conclude with discussion of overarching methods considerations.

## Motivating Example

People with SMIs like schizophrenia and bipolar disorder experience 10–20 years premature mortality relative to the overall US population (Olfson et al., 2015; Roshanaei-Moghaddam & Katon, 2009). This excess mortality is primarily driven by high prevalence of poorly controlled chronic health conditions, especially cardiovascular risk factors and cardiovascular disease. Multiple interrelated factors, including metabolic side effects of psychotropic medications and social risks for chronic disease like poverty and unemployment, contribute to the cardiovascular risk among people with SMI (Janssen et al., 2015). Further exacerbating the burden of chronic disease among this group is disconnection between the general medical system and the specialty mental health system, where many people with SMI receive services. Due in part to this system fragmentation, many people with SMI receive suboptimal preventive services and care for their co-morbid chronic physical health conditions (McGinty et al., 2015).

One approach to address this problem that is gaining traction in the USA is the “behavioral health home” model for physical health care coordination and management in mental health care settings, which was shown to improve preventive service use and quality of cardiometabolic care for people with SMI in randomized clinical trials (Druss et al., 2010, 2017). Key model components include systematic screening of the entire client panel and standard protocols for initiating treatment, use of a population-based registry to systematically track patient information and inform care, health education and self-management support for clients, care coordination and collaborative care management with physicians and other providers, and linkages with community and social services. Implementation is typically led by a nurse care manager.

Historically, lack of an insurance reimbursement mechanism to pay specialty mental health providers for delivery of “behavioral health home” services like physical health care coordination and management has been a key barrier to scaling the model. Starting in 2014, the Affordable Care Act’s (ACA) Medicaid health home waiver addressed this issue by allowing states to create Medicaid-reimbursed health home programs for beneficiaries with complex chronic conditions. As of October 2021, 19 states and Washington, D.C. had used the waiver to create behavioral health home programs for Medicaid beneficiaries with SMI (Centers for Medicare & Medicaid Services, 2020).

The ACA Medicaid health home waiver policy facilitated adoption of the behavioral health home model by creating a financing mechanism. However, studies suggest that additional implementation structures and strategies—for example, health IT infrastructure improvements, care team redesign, and provider training—are needed for the policy to support implementation of behavioral health home programs with fidelity to the model shown to be effective in clinical trials (Murphy et al., 2019). It is unknown which policy implementation structures and strategies need to accompany the ACA Medicaid waiver policy to support implementation of a behavioral health home model that will improve chronic disease care and outcomes for people with SMI.

## Objective and Definitions

### Objective

The expert forum focused on answering the question: what research methods can be used to study which policy implementation structures and strategies are needed to achieve policy goals? While we motivate this question around the ACA Medicaid health home waiver policy, the methods considerations are intended to apply to a range of policy scenarios. Policy changes frequently need a range of implementation actions in order to achieve their intended outcomes, especially policies designed to support scale-up of complex, multi-component interventions like behavioral health homes and other evidence-based chronic disease prevention and management interventions (Bullock et al., 2021).

### Definitions: Policy Implementation Structures, Strategies, and Outcomes

Policy implementation is broadly defined as translating a policy from paper to practice. We focus specifically on policies that are designed to lead to implementation of evidence-based health interventions or practices, like the behavioral health home program. We posit that policy implementation involves structures, strategies, and outcomes, as delineated in Table 1.

Policy implementation structures are the attributes of policies and policy-implementing systems and organizations that shape implementation. A state Medicaid health home waiver policy provision stating that a mental health program must have a behavioral health home medical director and nurse care manager on staff to bill Medicaid for health home services is an example of a policy implementation structure. We conceptualize policy implementation structures as including three broad sub-categories: provisions of the policy of interest, related policies, and system/organization environments. Policy provisions lay the groundwork for implementation of evidence-based practices, and the provisions of a single type of policy often differ across jurisdictions or organizations. For instance, the Medicaid reimbursement rate for behavioral health homes varies across states. While we are often interested in studying a single policy, like the ACA Medicaid health home waiver, that is designed to prompt implementation of an evidence-based based practice, other related policies can also influence implementation of that practice, a concept exemplified by Raghavan and colleagues’ policy ecology framework (Raghavan et al., 2008). The policy environment for behavioral health home implementation includes multiple types of policies, such as state behavioral health home licensing and accreditation policies, in addition to the Medicaid waiver policy (Stone et al., 2020). Finally, structural elements of the systems and organizations within which policies are implemented can influence policy implementation, for example staffing and health IT capacity. These three types of policy implementation structures are often interrelated: for example, a provision of many states’ health home waiver policies specifies the requisite staffing (a measure of organization-level structure) for behavioral health homes.

Policy implementation strategies are the methods or techniques used to put a policy into practice; examples in the ACA Medicaid health home waiver context are hiring, training, and coaching the behavioral health home nurse care manager in conducting evidence-based physical health care coordination and management for people with SMI. Strategies like training and coaching that can be used to implement policies can also be used to implement programs or practices in the absence of policies; “policy implementation strategies” are simply “implementation strategies” put in place in response to a policy. Implementation strategies and approaches for measuring these strategies have already been well characterized in the implementation science field (Powell et al., 2015; Leeman et al., 2017; Proctor et al., 2013). Our intent is not to suggest that different strategies are needed to implement policies designed to support the scale-up of evidence-based practices, but rather to point out that these policies need to be (but often are not) accompanied by effective implementation strategies.

Conceptually, the key policy implementation outcome is whether a policy achieved its intended goal. The challenges surrounding defining and building consensus around a policy’s goals are outside the scope of this article, but goal definition is critical to ascertaining a policy’s effectiveness (Meter et al., 1975). In our context, where we are focused on policies designed to support implementation of evidence-based practices, a policy goal can be thought of in terms of implementation (did the policy lead to uptake and implementation of the evidence-based practice by organizations and providers?), service receipt by the target population (did the policy help the people who need the intervention get it?) or health outcomes (did the people who got the intervention have improved health outcomes as a result?) (Proctor et al., 2011). Examples of each of these types of outcomes in the context of the Medicaid ACA health home waiver are included in Table 1.

## Approaches for Studying Policy Implementation

The expert panel viewed approaches for studying policy implementation in three general categories: descriptive, predictive or associational, or causal methods. The types of research questions that can be answered with different methods are delineated in Table 2 and discussed in more detail below.

### Methods that Aim to Describe or Document

Most studies of policy implementation to date have used descriptive approaches aiming to characterize policy implementation. While descriptive methods cannot answer questions about which implementation structures and strategies predict or cause policy implementation outcomes, they are often a critical first step to generating hypotheses. Descriptive approaches are commonly used to study policy implementation at a single point in time but can also be used to characterize changes over time. Descriptive study designs do not typically include a comparison group of non-policy adopting units, as the focus is on characterizing implementation in jurisdictions or organizations that implemented the policy.

Common methods include qualitative interviews with or surveys of policy implementers, review of documents relevant for implementation (e.g., agency guidance on how to comply with a policy), or descriptive quantitative analyses of secondary implementation data (e.g., logs of training dates, locations, and attendance). These methods, often in combination, have frequently been employed in case studies that detail policy implementation, often comparing across jurisdictions or organizations. Members of our author team (EEM, AKH, and GLD) used these methods to study implementation of Maryland’s behavioral health home model using a case-study approach that combined qualitative interviews and surveys with behavioral health home leaders and providers to characterize the policy implementation structures and strategies across organizations (Daumit et al., 2019; McGinty et al., 2018). There was considerable variation: for example, 39% of community mental health organizations implementing behavioral health home programs had either a co-located primary care provider or a formal partnership with a primary care practice (implementation structure) and 54% offered evidence-based practice trainings (implementation strategy).

Dimension reduction approaches such as latent class analysis, latent transition analysis, principal components analysis, and factor analysis are also potentially useful descriptive methods for studying policy implementation. These approaches can be used to understand what types of policy implementation structures and strategies cluster together. These methods have previously been used to characterize policy provisions, which, as noted above, shape implementation and often vary considerably across jurisdictions. Prior research led by a member of our author group (MC) has used latent class analysis to group state laws into classes of laws with similar provisions and used latent transition analysis to study the probability that state laws transitioned between classes over time (Cerdá et al., 2020; Martins et al., 2019; Smith et al., 2019). This approach could be extended to identify potential latent underlying constructs that connect different policy implementation structures and strategies and lead them to be clustered together. For example, in the context of the ACA Medicaid health home waiver, one could use these methods to identify similarities and differences across mental health clinics implementing a behavioral health home program regarding organizational structure and implementation strategies. Latent class analysis could be used to identify policy implementation classes, where clinics with similar implementation structures (e.g., co-located primary care providers and electronic health records) and implementation strategies (e.g., chronic disease management coaching and decision-support protocols) were clustered together. This is useful in that policy implementation structures and strategies are often not implemented in isolation, and certain combinations may often or always “go together.” In this situation, it is not informative to examine how individual implementation structures and strategies influence policy outcomes; rather, we want to study the clusters that occur in practice. Dimension reduction approaches can support identification of those combinations.

### Methods that Aim to Predict or Understand Associations

A set of methods—known as predictive or associational—go a step beyond descriptive approaches and aim to assess which implementation structures and strategies predict or are related to policy outcomes. Importantly, these methods are not trying—and cannot be used—to make causal inferences, as an unobserved policy implementation structure or strategy that is correlated with observed measures shown to predict a policy outcome might be the actual causal factor. However, these approaches can be informative in practice by predicting which jurisdictions or organizations are most likely to succeed with policy implementation using their existing structures/strategies and which might need additional support, or which factors might be worth causal investigation, discussed below. Like descriptive approaches, predictive or associational approaches for studying policy implementation likely do not include a comparison group of non-policy adopting units. Rather, the focus is on examining which implementation structures and strategies are related to policy outcomes among the subset of units implementing a policy.

Regression models are often used to understand associations between strategies and outcomes; they can be used to assess which policy implementation structures and strategies are strong and potentially statistically significant predictors of a policy implementation outcome of interest. As policy implementation often changes over time, occurs at multiple levels, and may be influenced by latent underlying constructs that may result in clusters of structures/strategies occurring together, regression modeling approaches that can handle these complexities, like hierarchical modeling and structural equation modeling, are often appropriate. To enhance generalizability of predictive regression models, cross-validation approaches can be used to ensure that a regression model predicting outcomes in one sample has similar results in a different sample (Picard & Cook, 1984). Regression models also have the advantage of having explainable and interpretable model forms.

Machine learning approaches such as random forests and simulated neural networks are an increasingly common predictive approach that could also be useful for studying policy implementation (Gates, 2017). These methods are particularly useful when there are many possible predictors, as is often the case in large administrative data sets, though there can be challenges with interpretation in these contexts. These approaches could be used to identify structures and strategies—and combinations thereof—that most reliably predict which jurisdictions or organizations achieve desired outcomes. For example, random forest modeling uses a collection of decision trees to predict an outcome across multiple randomly selected bootstrapped samples. This approach has been used by one author (MC) to study how specific provisions of state laws designed to reduce opioid overprescribing predicted opioid dispensing in US counties (Martins et al., 2021). This and other “interpretable” machine learning methods could be similarly applied to study how varying policy implementation structures and strategies across jurisdictions/organizations predict policy outcomes.

In our ACA Medicaid health home waiver policy example, both regression and machine learning could be used to study which observed policy implementation structures and strategies, like the examples in Table 1, predict implementation, service delivery, and client outcomes. These predictive approaches have different strengths and could potentially be used in combination to compare findings. Regression modeling is focused on analyzing relationships among pre-specified variables and their interactions and uses confidence intervals, statistical significance tests, and other fit statistics to assess model performance and can yield interpretations of individual variable coefficients. Machine learning approaches often do not require pre-specification of functional forms and have greater predictive power than standard regression approaches; because of this they are particularly good at identifying complex and/or nonlinear relationships, but they are often less interpretable in that they do not provide coefficients for individual variables but rather just identify the strongest predictors. For example, random forests can be used to predict an outcome and identify a set of variables—and their interactions—important for those predictions, but they do not readily admit clear explanations of the relationships between variables and outcomes. Thus, regression modeling and machine learning are not clearly distinct approaches; they have similar goals, but different strengths and limitations as discussed above.

Systems science approaches such as agent-based modeling and systems dynamics modeling may also be useful approaches for studying associations relevant to policy implementation (Langellier et al., 2019). Policy implementation occurs within complex and dynamic health systems, with interdependence and feedback across elements. Systems approaches have the potential to model how policy implementation structures/strategies might interact with other elements of the system to influence policy outcomes. One class of system approaches, known as system dynamics, uses a participatory research approach in model development (Hovmand, 2014). Stakeholders are engaged, typically using a scripted protocol (Calhoun et al., 2010), to provide qualitative information by identifying key factors and informing the conceptual model structure (Siokou et al., 2014; Weeks et al., 2017). The resulting model is subsequently used to guide data collection and quantitative model development (Haroz et al., 2021; Links et al., 2018). For example, agent-based modeling, another important class of system approaches, has been used by one of our authors (MC) to study how alcohol taxes influence violent victimization in New York City (Keyes et al., 2019). To do this, the modelers used empirical data including nonexperimental causal inference studies quantifying the effects of alcohol taxes on alcohol consumption to calibrate (by comparing agent-based-model estimates to empirical estimates) the model. Data and measurement challenges are a key limitation of these models, which need to be “parameterized” with data from other studies (Huang et al., 2021). Bayesian techniques for calibrating system science models, such as the Approximate Bayesian Computation method, have emerged as efficient tools that integrate simulation with prior information on uncertain model parameters (Pritchard et al., 1999).

### Methods that Aim to Examine Causal Links

Another set of methods aim to explain the causes of phenomena—not just associations. When designed and executed well, and when their underlying assumptions are satisfied, these types of studies can tell us which policy implementation structures and strategies operate as causal mechanisms in achieving policy outcomes. Methods aiming to establish causality require establishment of temporality, where policy implementation structures and strategies are in place and measured prior to policy outcomes. These approaches to studying policy implementation often involve a comparison group of non-policy adopting units or a comparison of groups that implement the policies in different ways. The former study design allows us to answer questions like “Relative to jurisdictions without the policy, did jurisdictions with policy implementation structure/strategy set A lead to desired policy outcomes?” whereas the latter study design allows us to answer questions like “Relative to policy implementation structure/strategy set A, does structure/strategy set B lead to desired policy outcomes?”.

However, such comparisons are challenging because entities that do and do not implement a policy, or implement in a certain way, likely differ from each other in other ways. Randomization is one approach to avoid that situation, known as confounding, but is generally implausible in the policy adoption context (though not impossible, as shown in several prominent examples (Baicker et al., 2013; Ludwig et al., 2008)). Rather, it is more feasible to randomly assign timing of policy implementation across jurisdictions or organizations or to conduct randomized experiments comparing two (or more) different policy implementation strategies. For example, mental health clinics creating behavioral health home programs as a result of their state’s adoption of the ACA Medicaid health home waiver policy could be randomly assigned to different implementation strategies: half the clinics might be randomly assigned to receive training for providers, and the other half might be randomly assigned to receive training for providers, plus ongoing provider coaching, plus resources and technical assistance to build an electronic patient registry.

Nonexperimental methods for causal inference are approaches that use natural experiments to examine potentially causal factors, like our ACA Medicaid health home waiver example, in which there is variation in policy adoption across units and over time (West et al., 2008) (Table 3). In our case, 19 states and D.C. used the ACA waiver to create behavioral health home programs for Medicaid beneficiaries with SMI between 2014 and 2021, and 31 states did not; in addition, many states allowed mental health organizations to opt-in to the waiver-created program, resulting in variation in policy adoption across organizations within states. Both cases result in scenarios where some units (states or organizations) have the policy and others do not, and both set-ups have been used to study the outcomes of the ACA health home waiver in prior research (Bandara et al., 2019; Murphy et al., 2019; McClellan et al., 2020; McGinty et al., 2019).

Nonexperimental approaches for causal inferences like difference-in-differences and augmented synthetic controls (Ben-Michael et al., 2018) capitalize on the ability to measure trends in outcomes of interest before and after a policy was implemented in policy-adopting and comparison states to make causal inferences. To apply these approaches to study of policy implementation, it may be important to operationalize the independent policy variable(s) to indicate degree of policy implementation (either overall or with respect to specific policy provisions). For example, rather than defining the policy variable to reflect implementation status (yes/no) of a state with respect to the ACA waiver-behavioral health home program, one could alternatively define the policy variable as an indicator of implementation robustness (e.g., measured on a continuous scale). A less common approach with potentially useful applications in policy implementation that also aims to establish causal links is configurational analysis (Whitaker et al., 2020). This case-based method uses Boolean algebra and set theory to identify “difference-making” combinations of conditions that uniquely distinguish one group of cases with an outcome of interest from another group without that same outcome. Cases, in the policy implementation context, are policy-implementing units, for example mental health programs implementing behavioral health homes through their state’s Medicaid health home waiver. Equifinality, or the idea that multiple bundles of conditions can lead to the same outcome, is a key property of configurational analysis. Thus, in contrast to variable-oriented approaches, configurational analyses can yield multiple solutions—in other words, multiple combinations of policy implementation structures/strategies—that produce the same result. This property is potentially useful as it can provide context-sensitive results as well as give decision makers multiple options to choose from when designing policy implementation in their jurisdiction or organization. In a simplistic example, imagine we have categorized the 29 state ACA Medicaid health home waiver policies as follows, based on their provisions: policies with high vs. low reimbursement rate, policies with robust versus limited staffing requirements, and policies with strong versus weak performance monitoring requirements. The results of a configurational analysis might show that state waiver policies that either had high reimbursement alone *or* that had robust staffing + strong performance monitoring requirements had improvements in quality of cardiovascular care for people with SMI.

A challenge for studies aiming to estimate causal links is that they often involve substantial assumptions to get around the challenge that we do not get to observe the causal links of interest. We only see sites, for example, implement a policy in a particular way—we cannot also directly observe their outcomes had they implemented differently. Given inherent confounding and potential differences between individuals who receive different levels of intervention in non-experimental contexts, a challenge with any non-experimental study is that untestable assumptions will be required to interpret effect estimates as causal. Different designs use a variety of assumptions—such as that of no unmeasured confounding in propensity score comparison group designs—to estimate causal effects, and it is crucial to interpret study results within the context of the reasonableness of the underlying assumptions in that study. A detailed discussion of the assumptions of a range of non-experimental study designs, and strategies for minimizing those threats, is outside the scope of this paper but available elsewhere (Schuler et al., 2021). The policy implementation context involves further challenges, such as the multilevel nature of many of the research settings. Given their underlying and untestable assumptions, once methods that aim to explain causal links are extended to these complex settings, care must be taken with their use and the interpretation of study results.

## Priorities for Advancing Policy Implementation Research Methods

A key conclusion of our expert forum was that existing methods could be used more frequently to study policy implementation—as discussed above—and that additional methodological innovation in approaches for studying policy implementation are needed. Specifically, we identified three priorities: (1) effect modification methods for making causal inferences about how policies’ effects on outcomes vary based on implementation structures/strategies; (2) causal mediation approaches for studying policy implementation mechanisms; and (3) characterizing uncertainty in systems science models. We describe each of these in more detail below.

### Effect Modification Approaches

Examining how the effects of a policy on outcomes differ depending upon implementation structures and strategies is conceptually an effect modification question: does the presence/absence of policy implementation structures/strategies modify the effects of the policy on outcomes? For example, one might ask whether mental health clinics with electronic medical record systems were more successful at implementing behavioral health home programs through their state’s ACA waiver policy than those without such systems. The challenge is that traditional effect modification approaches require that the “modifier”—the policy implementation structure/strategy in our case—be present in both the treatment and the control group, and that it be measured at baseline. This condition may be met for some implementation structures that are a characteristic of the implementation setting and pre-date the policy (e.g., an electronic medical record system was in place at a mental health clinic before that clinic used the ACA waiver to create a behavioral health home program), but it is not met for implementation structures and strategies that are put in place following policy adoption (e.g., a mental health clinic that created an electronic medical record system as part of its behavioral health home program implementation efforts).

In nonexperimental settings, there are a variety of approaches to investigate effect modification of a set of pre-policy characteristics of the implementation settings. Stratification is a traditional method for examining effect modification. In stratified analyses, the policy effect is estimated separately among groups of units using the same implementation strategy/structure. For example, we could separately evaluate the effects of a state’s Medicaid health home waiver policy on adoption of the behavioral health home program in two groups, or strata, of mental health clinics: those with an electronic medical record system in place prior to the policy and those without. We could then estimate and compare the effect of the policy on behavioral health home adoption in both strata, although stratification does not allow formal testing of whether the effect varies across strata. To do that, we can use a regression framework, including treatment-by-implementation-strategy/structure interaction terms, which allows formal testing of whether the effect of the policy on the outcome of interest varies across strata.

Stratification and regression can also be combined in a two-step procedure in which first the effects of the policy are estimated separately for each site, then the estimated policy effects are regressed on the site-specific implementation strategies/structures. This approach, which is conceptually similar to meta-regression of clinical trial effect estimates on study-level covariates in meta-analysis, can yield estimates of associations between specific implementation strategies/structures and policy effect size. For example, the effects of the behavioral health home on quality of cardiovascular care could be estimated for each organization that adopted its state’s health home policy. These effects could then be regressed on implementation strategies/structures that were in place at those organizations at the time of policy adoption (Kennedy-Hendricks et al., 2018) and that might explain some of the heterogeneity in health home effects across organizations.

In addition to standard effect modification approaches like those mentioned above, we may also be able to borrow methods from the literature on individualizing treatment. Adaptive implementation strategies can recommend different implementation structures/strategies to different policy-implementing units based on characteristics that may change over time (Kilbourne et al., 2013). These may be of particular interest when considering *sequences* of implementation structures/strategies that may work well in some settings and less well in others. Consider again the Medicaid health home waiver example. An adaptive implementation strategy for mental health clinics using their state’s waiver to implement behavioral health home programs might start with two months of training for all clinics. For some clinics, this training will be sufficient to achieve high-fidelity implementation, defined as the clinic’s behavioral health home program including the same elements as the model programs shown to improve patient care and health outcomes in clinical trials. Following the training, these clinics do not need additional implementation support. However, for other clinics, this two-month training will not be sufficient to achieve high-fidelity behavioral health home implementation. For these clinics, the adaptive implementation strategy might involve following up with two months of expert coaching, and then re-assessing fidelity to see if additional implementation support is needed. The adaptive implementation strategy recommends an initial approach, monitors’ implementing units’ (clinics, in this example) performance, and modifies implementation support based on that monitoring.

High-quality adaptive implementation strategies can be discovered using either experimental or non-experimental approaches. If it is feasible to randomize policy-implementing units to different implementation strategies, the sequential multiple-assignment randomized trial (SMART) may be useful (Kilbourne et al., 2014; Murphy, 2005). Alternatively, machine learning methods such as Q-learning have been adapted to make causal inference for adaptive implementation strategies using nonexperimental data (Moodie et al., 2012). In either case, the goal is to use data to learn about how to construct an effective adaptive implementation strategy that can then be rolled out to other jurisdictions.

### Causal Mediation Methods

Policy implementation structures/strategies might, in some cases, be on the causal pathway from policy adoption to policy outcome, in which case they would be appropriately analyzed as mediators, rather than effect modifiers. For example, a key element of behavioral health home implementation is hiring a nurse care manager to lead implementation efforts. In a scenario where reimbursement for behavioral health services finances the nurse’s salary, hiring that nurse care manager is on the causal pathway from adoption of the Medicaid waiver to all policy outcomes, including implementation outcomes (e.g., fidelity of behavioral health home program implementation), service outcomes (e.g., quality of care for people with SMI), and health outcomes (e.g., cardiovascular risk factor control among people with SMI). However, studying causal mediation is very challenging because in addition to baseline confounding that is almost always present in non-experimental studies, there may be confounding due to factors affected by the policy, known as “post-treatment confounding.” For example, health homes may hire a nurse care manager in part because they are or are not seeing good outcomes after implementing the policy, which will complicate studies aiming to examine the mediating effect of hiring a nurse care manager (Nguyen et al., 2021; Stuart et al., 2021). An additional challenge in the policy implementation context is that existing mediation analysis methods may need to be adapted for the small sample size often available for policy implementation research.

### Characterizing Uncertainty in Systems Science Models

Data and measurement challenges are a key limitation of systems science models, which usually need to be “parameterized” with data from other studies. While this type of systems approach could be useful for studying policy implementation given that interactions among implementation structures and strategies and unexpected elements within complex systems are often key drivers of implementation success/failure, using systems approaches to accurately explain which policy implementation structures/strategies are associated with policy goals requires robust empirical analyses using other methods. Systems models may be most valuable in hypothesis-generating scenarios where we want to explore how parts of complex systems may interact with one another, through feedback loops and other nonlinear relationships, to influence policy implementation and outcomes.

It is also crucial for the results of the models to reflect the uncertainty that exists—uncertainty regarding the data and data sources, statistical uncertainty due to parameter estimation, and structural uncertainty in terms of, for example, which elements of the system are included in the model (D’Agostino McGowan et al., 2021). An additional challenge is that in some cases the parameter values are coming from locations or groups that may differ from the specific policy implementation context being modeled—for example, information on health care service utilization among a commercially insured population compared to a publicly insured population.

### Final Considerations

Our expert forum identified three overarching considerations around measurement, context, and causality. As noted in the introduction to this piece, studying policy implementation using any of the methods discussed above requires valid and reliable measurement of implementation structures and strategies. While multiple implementation measurement frameworks exist, none of them are specific to policy implementation; such a framework could help standardize policy implementation measurement in the field. Context is critical in studying policy implementation: the implementation structures/strategies needed for a policy to achieve its goals likely often depend upon context, e.g., large versus small health systems, urban versus rural jurisdictions. While it is often infeasible to conduct randomized experiments in all possible contexts, nonexperimental methods that aim to document, describe, predict, and examine causal links for policy implementation research are well suited for studying context (Alegria, 2022). In research assessing causal links, context often can be analyzed using standard effect modification approaches, although some analyses may be limited by relatively small sample sizes (especially of state policies) and thus limited ability to disentangle relationships. Finally, it is critical to consider when determining causality is the goal of policy implementation research. In many cases an understanding of what factors relate to better outcomes—examined through descriptive or predictive approaches—will be an important step and may spur additional policy implementation innovation and further research. In conclusion, simply enacting a policy is typically not enough to spur achievement of the policy’s goals: implementation is critical. Applying existing methods in innovative ways and developing new methods to better study policy implementation is critical to widely scaling evidence-based health interventions.

## Figures and Tables

**Table 1:** Key components of policy implementation

Policy Implementation Structures & Strategies	Definition	Example in ACA Medicaid waiver behavioral health home context
Policy Implementation Structures	Attributes of policies and policy-implementing systems that affect implementation	See structure sub-categories below
Provisions of the policy of interest	The specific rules laid out in the policy that affect implementation	Waiver policy provisions defining the required staffing composition and staff-to-client ratio for behavioral health home programs
Related policies	Other policies at multiple levels of government that shape implementation of the policy of interest	State Medicaid agency policies regarding behavioral health home licensing and accreditation
System & organization environment	Structural elements of policy-implementing systems and organizations	ACA-waiver behavioral health home staffing and health IT capacity, e.g., presence of an electronic health record system
Policy implementation strategies	On-the-ground techniques used to translate a policy into practice, including implementation and enforcement activities	Chronic disease management training, coaching, and decision-support for mental health providers practicing in ACA-waiver behavioral health home programs
Policy implementation outcomes	Indicators of the degree to which the policy met its implementation, service delivery, and population health goals	See outcome sub-categories below
Implementation outcomes	Degree to which the policy supported uptake and implementation of the evidence-based practice by organizations/providers	Proportion of eligible mental health organizations that used a state’s Medicaid health home waiver to implement a behavioral health home program
Service outcomes	Whether the policy led to receipt of the evidence-based practice by people in need	Quality of cardiovascular care for people with SMI
Health outcomes	Whether the policy led to improvements in the health outcome(s) targeted by the evidence-based practice	Cardiovascular risk factor control among people with SMI

**Table 2:** Policy implementation research methods

Policy Implementation Research Methods	Example Research Questions
**Methods that aim to describe or document**	
Qualitative research	What structures and strategies do leaders of ACA-waiver behavioral health home programs think are needed for successful implementation and why?
Survey research	What policy implementation strategies do ACA-waiver behavioral health home providers report using at their clinic?
Document review	What are the provisions of each state’s ACA Medicaid health home waiver policy that could influence behavioral health home implementation?
Case studies	How do the answers to the three questions above vary in state A versus state B?
Descriptive analyses of implementation	How many chronic care management trainings were conducted for mental health providers implementing ACA-waiver behavioral health home programs?
Dimension reduction approaches, e.g., latent class analysis	Across the 250 community mental health programs in a single state that are implementing a behavioral health home program through their state’s ACA waiver, are there clusters of organizations with similar policy implementation structures/strategies?
**Methods that aim to predict or understand associations**	
Regression approaches	Which policy implementation structures and strategies are statistically significant predictors of a mental health clinic’s ability to implement an ACA-waiver behavioral health home with high fidelity?
Machine learning approaches, e.g., random forests	Can we reliably predict which measured implementation structures and strategies predict high-fidelity ACA-behavioral health home implementation by a mental health clinic?
Systems science methods, e.g., agent based modeling	How do policy implementation structures and strategies interact with other features of the complex health system to influence fidelity of ACA-waiver behavioral health home implementation?
**Methods that aim to examine causal links**	
Randomized experiments	Which policy implementation structures and strategies cause high-fidelity ACA-waiver
Nonexperimental approaches for causal inference, e.g., difference-in-differences	behavioral health home implementation?
Configurational analysis	Which combinations of policy implementation structures and strategies uniquely distinguish mental health clinics with high- versus low-fidelity of ACA-waiver behavioral health home implementation?

**Table 3:** A small sampling of nonexperimental approaches to causal inference

Approach	Description	Key Assumptions or Limitations
Interrupted time series	In the population of people responsible for implementing the policy, extrapolate pre-policy outcome trends into the post-policy period to predict what would have happened without the policy, then compare with the observed post-policy outcomes (Kontopantelis et al., 2015)	*Parallel trends:* In the absence of the policy, the post-policy outcome trajectory would be the same as it was pre-policy, i.e., we can extrapolate the pre-policy trends into the post-policy period *Lack of comparison group:* Because there is no untreated group, comparisons are only as good as the model used for extrapolation of the pre-policy outcomes into the post-policy period
Difference-in-differences (with variations that include event studies, trial emulation, comparative interrupted time series; (Roth et al., 2022)	Estimates the difference in average outcomes between pre- and post-policy periods in both treated and untreated groups, then compares the difference in those differences. Sometimes also models and compares the trends in outcomes across the groups, not just the levels pre- and post, commonly using regression techniques. Appropriate untreated comparison groups can be constructed using (augmented) synthetic controls or other approaches (Ben-Michael et al., 2021).	*Parallel trends:* In the absence of policy, the outcome trajectory in the treated group would have changed in the same way pre to post as did the outcome trajectory in the comparison group *Staggered adoption:* When treated units are treated at different times, estimating average policy effects across units requires more complex methods; see, e.g., (Callaway et al., 2021)
Regression discontinuity	Estimates a causal effect using discrete fixed-by-design cutoffs in some variable or characteristic to determine which units receive policy. Units just above and just below the cutoff are compared (Linden et al., 2003).	*Smoothness:* Units just above and just below the cutoff should be essentially similar; the outcome-generating mechanism is similar above and below the cutoff, i.e., outcomes from units above and below the cutoff are “comparable”; the variable used to define the cutoff wasn’t manipulated to manipulate which units receive policy *Known assignment mechanism:* The cutoff point for determining which units are treated must be known in advance
Instrumental variables	Uses an “instrument” that is related to policy but not directly related to the outcome, and which can be thought of as randomly assigned. Estimation generally uses a two-stage least squares approach that models the policy of interest as a function of the instrument, and the outcome as a function of the predicted policy received (from that first stage; (Hernán & Robins, 2006).	*Finding good instruments can be difficult:* The requirements of “random assignment” and lack of direct effects on the outcome are untestable and rely on sound scientific arguments *Local average treatment effect:* Instrumental variable approaches estimate the causal effect of policy among those individuals for whom the instrument determines their policy exposure
Sample equating approaches	A class of strategies (including matching, weighting, and subclassification) that manipulate the sample such that the treated and untreated groups are similar on observed variables. This includes propensity score and matching approaches, among others (Stuart, 2010).	*No unmeasured confounders:* There are no confounding (or “lurking”) variables other than those included in the propensity score model or on which units are being matched *Common support:* There are treated and untreated units across the range of the other group’s covariate distribution
Marginal structural models	Similar to sample-equating approaches, but for time-varying confounding and time-varying exposures. Inverse probability-of-treatment weights are constructed separately for every unit-time point, then a series of weighted regression models are fit to the data. (Shinozaki & Suzuki, 2020).	*No unmeasured confounders:* See above
Configurational analysis	Identifies “difference-making” combinations of conditions that distinguish between groups of units with different outcomes (Whitaker et al., 2020).	*No unmeasured confounders:* See above *Model-based:* Depends on correctly specifying possible complex causal pathways and relationships between covariates *Positivity:* Can only discover difference-making configurations that are present in the data; will likely not uncover full causal structure
