# Correction to: Scaling Interventions to Manage Chronic Disease: Innovative Methods at the Intersection of Health Policy Research and Implementation Science

**DOI:** 10.1007/s11121-023-01569-3

**Published:** 2023-07-03

**Authors:** Emma E. McGinty, Nicholas J. Seewald, Sachini Bandara, Magdalena Cerdá, Gail L. Daumit, Matthew D. Eisenberg, Beth Ann Griffin, Tak Igusa, John W. Jackson, Alene Kennedy-Hendricks, Jill Marsteller, Edward J. Miech, Jonathan Purtle, Ian Schmid, Megan S. Schuler, Christina T. Yuan, Elizabeth A. Stuart

**Affiliations:** 1 Department of Health Policy and Management, Johns Hopkins Bloomberg School of Public Health, Baltimore, MD USA; 2 Department of Mental Health, Johns Hopkins Bloomberg School of Public Health, Baltimore, MD USA; 3 Department of Population Health, New York University Grossman School of Medicine, New York, NY USA; 4 Division of General Internal Medicine, Johns Hopkins School of Medicine, Baltimore, MD USA; 5 RAND Corporation, Washington, DC USA; 6 Department of Engineering, Johns Hopkins University, Baltimore, MD USA; 7 Department of Epidemiology, Johns Hopkins Bloomberg School of Public Health, Baltimore, MD USA; 8 Indiana University School of Medicine, Indianapolis, USA; 9 Department of Public Health Policy and Management, New York University School of Global Public Health, New York City, NY USA

**Correction to: Prevention Science** **https://doi.org/10.1007/s11121-022-01427-8**

The article “Scaling Interventions to Manage Chronic Disease: Innovative Methods at the Intersection of Health Policy Research and Implementation Science”, written by McGinty, E.E., Seewald, N.J, Bandara, S., Cerdá, M., Daumit, G.L., Eisenberg, M.D., Griffin, B.A., Igusa, T., Jackson, J.W., Kennedy‑Hendricks, A., Marsteller, J., Miech, E.J., Purtle, J., Schmid, I., Schuler, M.S., Yuan, C.T., and Stuart, E.A., was originally published electronically on the publisher’s internet portal on 01 September 2022 without open access. With the author(s)’ decision to opt for Open Choice the copyright of the article changed on 07 June 2023 to © The Author(s) 2023 and the article is forthwith distributed under a Creative Commons Attribution 4.0 International License, which permits use, sharing, adaptation, distribution and reproduction in any medium or format, as long as you give appropriate credit to the original author(s) and the source, provide a link to the Creative Commons licence, and indicate if changes were made. The images or other third party material in this article are included in the article’s Creative Commons licence, unless indicated otherwise in a credit line to the material. If material is not included in the article’s Creative Commons licence and your intended use is not permitted by statutory regulation or exceeds the permitted use, you will need to obtain permission directly from the copyright holder. To view a copy of this licence, visit http://creativecommons.org/licenses/by/4.0/.

The original article has been corrected.

